# Electrochemical Sensing of Dopamine with P-g-C_3_N_4_/ZIF-67/CPE Composite Electrodes

**DOI:** 10.3390/bios16040224

**Published:** 2026-04-18

**Authors:** Yan Deng, Yixin Liao, Teresa Murray, Shengnian Wang

**Affiliations:** 1 Institute for Micromanufacturing, Louisiana Tech University, Ruston, LA 71272, USA; 2 Center for Biomedical Engineering and Rehabilitation Sciences, Louisiana Tech University, Ruston, LA 71272, USA; tmurray@latech.edu

**Keywords:** metal–organic framework (MOF), ZIF-67, carbon nitride, electrochemical sensor, dopamine

## Abstract

Dopamine is a key neurotransmitter and neuromodulator that regulates many critical brain functions. Accurate monitoring of its level is essential for neuroscience as well as the diagnosis and treatment of many brain diseases. In this work, we developed a new electrochemical sensor, comprising phosphorus-doped graphitic carbon nitride (P-g-C_3_N_4_) and zeolitic imidazolate framework 67 (ZIF-67), for dopamine detection. In this composite electrode material, ZIF-67 provides numerous adsorption and sensing sites, while P-g-C_3_N_4_ enhances overall electrical conductivity and stability. Cyclic voltammetry tests reveal the redox behavior of dopamine at the surface of the composite electrode across various pH values and scan rates. Using differential pulse voltammetry, the sensitivity and selectivity of this dopamine sensor were assessed, identifying a limit of detection of 0.39 nM. Further successful quantification of dopamine in urine samples suggests the potential practical use of this new composite electrochemical sensor for detecting dopamine and/or other neurotransmitters.

## 1. Introduction

Dopamine is a vital excitatory neurotransmitter and neuromodulator in the central nervous system of mammals [1,2]. This phenethylamine and catecholamine compound regulates physiological functions in various human systems such as the central nervous system, renal system, and hormonal systems. The imbalance of the dopamine level could lead to many diseases, such as Parkinson’s disease, attention deficit hyperactivity disorder, Tourette syndrome, schizophrenia, and bipolar disorder [3,4,5,6,7,8,9]. Therefore, fast and accurate determination of dopamine concentration is critical for understanding and maintaining the physiological functions of the brain and other body parts involved in attention, learning, memory, movement, mood, behavior, and cognition [10,11,12].

Electrochemical sensors have been well studied for dopamine detection over the past several decades [13]. Different electrode modifiers, including metal and metal oxide nanomaterials (metal nanostructures and metal oxide nanostructures), carbon materials (carbon nanotubes, graphene), and polymer materials (conducting polymers, molecularly imprinted polymers), have been exploited to improve the sensitivity, selectivity, and/or robustness of the dopamine sensors [14,15,16,17,18]. Given the limitations of individual sensing materials, composite electrodes are often preferred for their large surface area, abundant sensing sites, excellent conductivity, and mechanical robustness, thereby enhancing sensitivity and/or selectivity for dopamine sensors [19,20,21,22,23,24,25,26,27].

In this study, a new composite modifier comprising phosphorus-doped graphitic carbon nitride (P-g-C_3_N_4_) and a cobalt-based metal–organic framework (MOF) was used to modify carbon paste electrodes for the detection of dopamine. MOFs are crystalline materials composed of metal clusters coordinated with organic ligands [28]. Owing to their huge surface areas, tunable structures, and rich active sites, MOFs are widely used for gas storage [29], water capture [30], energy storage [31], and sensor applications [32]. One specific MOF, namely ZIF-67, is used as a key component in this new electrochemical sensor. ZIF-67 is a cobalt-based zeolitic imidazolate framework (ZIF), a major class of MOFs, with a high surface area and microporosity [33,34]. The cobalt centers in ZIF-67 that coordinate to 2-methylimidazole in its structure can catalyze dopamine oxidation at the electrode surface, thereby boosting the redox peak current and improving the sensor’s sensitivity [35]. P-g-C_3_N_4_ is included to improve the mechanical properties [36] and electrical conductivity [37,38] of the composite sensor. Cyclic voltammetry (CV) was first carried out to investigate the redox behavior of dopamine on the surface of the new nanocomposite electrode material (P-g-C_3_N_4_/ZIF-67) under various electrochemical conditions (e.g., pH, scan rate). Using differential pulse voltammetry (DPV), the sensitivity and selectivity of this electrochemical sensor were then evaluated in the presence of individual interferences and their combinations. Further testing of urine samples paves the way for the use of this new composite electrode in practical neurotransmitter detection.

## 2. Experimental

### 2.1. Materials

Except for graphite powder from Merck & C (Rahway, NJ, USA), all other chemicals used in this study were purchased from Sigma-Aldrich (St. Louis, MO, USA). Among them, melamine, cobalt(II) nitrate hexahydrate (Co(NO_3_)_2_·6H_2_O), phosphoric acid, 2-methylimidazole (C_4_H_6_N_2_) were received as reagent grade. Dopamine (DA), serotonin (5-HT), ascorbic acid (AA), and uric acid are in analytical grade. All reagents were used as received unless specified.

### 2.2. Synthesis of ZIF-67

ZIF-67 was synthesized according to a procedure described in our previous work [39]. In brief, 12 mL of Co(NO_3_)_2_·6H_2_O (64.2 mM) and 80 mL of C_4_H_6_N_2_ (9.5 mM) were mixed for 6 h under vigorous agitation. The solution was then transferred to a stainless-steel autoclave and incubated there at 60 °C for 24 h. After washing with methanol, the purple precipitate was collected by centrifugation and dried (designated as ZIF-67).

### 2.3. Synthesis of Phosphorus-Doped Graphitic Carbon Nitride (P-g-C_3_N_4_)

To synthesize P-g-C_3_N_4_, melamine was added to a 2% (*V*/*V*) aqueous phosphoric acid solution (100 mL), and the mixture was stirred for 1 h. The filtered precipitation was washed with deionized water and calcined at 550 °C for 1.0 h to obtain P-g-C_3_N_4_.

### 2.4. Material Characterization

The functional groups in the received samples were recorded using a Fourier transform infrared (FTIR) spectrometer (Thermofisher Nicolet Avatar 330, Waltham, MA, USA). The sample morphology was examined using field-emission scanning electron microscopy (FE-SEM; Hitachi S-4800, Tokyo, Japan). Energy-Dispersive X-ray Spectroscopy (EDS) was performed using a Bruker EDS module (XFlash 6160, Billerica, MA, USA) installed on the SEM. Wide-angle X-ray diffraction (XRD) was performed using an XPertPro diffractor (Malvern Panalytical, UK) with Cu Kα radiation (λ = 1.54 Å). Nitrogen adsorption isotherms were obtained using a Surface Area and Porosity Analyzer (NOVA 2020e, Micromeritics, Norcross, GA, USA) after degassing at 150 °C.

### 2.5. Preparation of the Electrochemical Sensor

The working electrodes were prepared by mixing an optimized amount of P-g-C_3_N_4_/ZIF-67 composite with graphite powder and paraffin oil (as the binder). The received paste was then loaded into the end of a holder (0.3 cm in diameter) with a coaxial cable inserted. The unmodified carbon paste electrode was prepared by using only graphite powder and paraffin oil.

### 2.6. Electrochemical Measurements

All electrochemical tests were performed on an Autolab potentiostat (PGSTAT302N, Metrohm, Herisau, Switzerland). For voltametric experiments, a 3-electrode configuration was used with the carbon paste electrode (CPE) as the working electrode, a platinum wire as the counter electrode, and a saturated calomel electrode as the reference electrode. Electrochemical impedance spectroscopy (EIS) tests were conducted in 5 mM [Fe(CN)_6_]^3−/4−^ solutions under the following conditions: a frequency range of 10^−1^ to 10^5^ Hz; a potential of 0.24 V; an amplitude of 0.01 VRMS. Cyclic voltammograms (CVs) of the modified and unmodified electrodes were recorded in 0.1 M phosphate buffer solutions (10 mL) with a voltage range of −0.25 to 1.2 V. Differential pulse voltammetry (DPV) was done with a pulse amplitude of 50 mV and pulse width of 40 ms at a scan rate of 60 mV s^−1^. All tests were performed in triplicate (***n*** = 3) unless specified.

The sensitivity (***S***) of the electrochemical sensor was calculated from the gradient of the linear portion of the concentration calibration curve as:

***S*** = **Δ*I***/**Δ*C*** (1)

where **Δ*I*** is the change in current (in µA), and **Δ*C*** is the change in concentration (in nM). The limit of detection (***LOD***) was calculated as:

***LOD*** = k × ***σ***/***S*** (2)

where ***σ*** is the standard deviation of the blank samples (i.e., noise level), ***S*** is the sensitivity calculated using Equation (1), and k is the confidence level parameter (set as 3 in the ***LOD*** calculation). Similarly, the level of quantification (***LOQ***) was calculated using the same formula, with k set to 10.

## 3. Results and Discussion

### 3.1. Characterization of P-g-C_3_N_4_/ZIF-67 Composites

**Figure 1:**
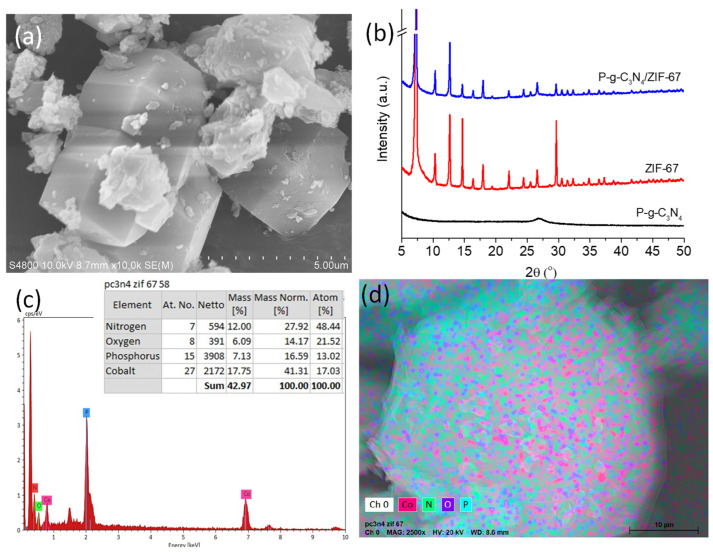
The SEM image (**a**), XRD (**b**), and EDS spectra (**c**) and element mapping (**d**) of P-g-C_3_N_4_/ZIF-67 composites. The XRD spectra of P-g-C_3_N_4_ and ZIF-67 alone are also provided in panel b for comparison purposes.

The morphology of the synthesized P-g-C_3_N_4_/ZIF-67 composites was first characterized by SEM, XRD, and EDS. As shown in Figure 1a, crystals with the classical rhombic dodecahedral shape were found in the SEM image, confirming the successful synthesis of ZIF-67. Those crystals exhibit sharp edges and well-defined facets, with an average grain size of ~5 µM. Small P-g-C_3_N_4_ nanoparticles attach to the surface of ZIF-67 crystals, while some large particles agglomerate around them. For the large P-g-C_3_N_4_ microparticles, a stacked thin-sheet structure is clearly visible.

The crystal structures of P-g-C_3_N_4_, ZIF-67, and P-g-C_3_N_4_/ZIF-67 were further revealed by their XRD spectra. As shown in Figure 1b, P-g-C_3_N_4_ exhibits a peak at 2***θ*** = 27.5°, which represents the (002) plane of carbon materials. The (100) plane peak of g-C_3_N_4_ (2***θ*** = 13.7°) is almost undetectable due to the low crystalline degree after heavily doping phosphorus atoms. All the diffraction peaks of ZIF-67, both the intensive reflection at 7.3° (110), 10.3° (200), 12.7° (211), 14.7° (220), 16.4° (310), 18.0° (222), and higher-order sodalite reflection peaks at 22.2°, 24.5°, 26.6°, 29.7° and beyond, are well preserved after the formation of the P-g-C_3_N_4_/ZIF-67 composites [27]. The (002) plane peak from P-g-C_3_N_4_ results in a typical graphitic hump in the XRD spectrum of the composites around 26–30°. Together, these results confirm the successful preparation of P-g-C_3_N_4_/ZIF-67 composites.

The EDS analysis of the P-g-C_3_N_4_/ZIF-67 composites confirms the existence of Co, O, and C atoms from ZIF-67 besides the presence of N and P atoms from P-g-C_3_N_4_ (Figure 1c). The element-mapping results further demonstrate the uniform distribution of these atoms within individual composite particles (Figure 1d).

**Figure 2:**
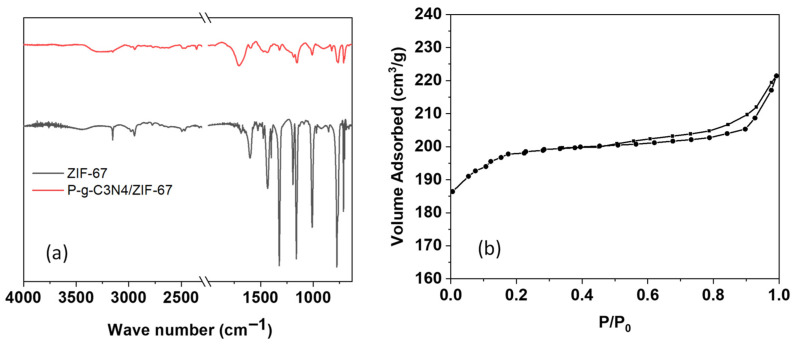
(**a**) The FTIR spectra of ZIF-67 alone and P-g-C_3_N_4_/ZIF-67 composites. (**b**) The N_2_ adsorption–desorption isotherms of P-g-C_3_N_4_/ZIF-67 composites. In panel b, data points with solid circles are from adsorption, and those with solid squares from desorption.

Given the importance of rich surface functional groups and the large surface area of electrode materials for sensing applications, we further characterized the received P-g-C_3_N_4_/ZIF-67 composites using FTIR spectroscopy and a surface area analyzer. Overall, most peaks in the FTIR spectra of ZIF-67 alone and P-g-C_3_N_4_/ZIF-67 composites are similar, with only slight differences observed (Figure 2a). The vibration peaks of aromatic C–H stretch of the imidazole ring (~3130–3150 cm^−1^) and that of aliphatic C–H stretch of the methyl group (~2920–2930 cm^−1^) stand out from the broad peak at the range of 2800–3500 cm^−1^ that is attributed to the presence of hydroxyl groups and N-H bond (at 3100–3300 cm^−1^). This confirms the presence of ZIF-67 in the composites. The adsorption bands that are assigned to C=C and C=N stretching in the imidazole rings are clearly seen at ~1580–1640 cm^−1^ (with a band near 1630–1640 cm^−1^ for C=C stretch and ~1580–1590 cm^−1^ for C=N stretch), as is the vibration band of aromatic C-N heterocycles at ~1680–1697 cm^−1^. The stretching vibration at 1640 cm^−1^, contributed by the carboxylic group and metal ions, largely overlaps with these two main bands. The absorption bands due to ring stretching display one peak at ~1313 cm^−1^ and another at ~1430 cm^−1^. The next band region at ~1140–1150 cm^−1^ is attributed to aromatic stretching. The peak at 810–850 cm^−1^ can be ascribed to the s-triazine cycle in P-g-C_3_N_4_. And the peaks observed at 1006 cm^−1^ are ascribed to the Co-O-C band, and the one at 765 cm^−1^ is ascribed to the substitution of Co on the benzene rings in ZIF-67. The Co–imidazolate coordination gives rise to a vibrational band at ~700–710 cm^−1^. The FTIR results support the formation of the ZIF-67 framework, and the addition of P-g-C_3_N_4_ retained their function groups in the composites.

The N_2_ physical adsorption was also performed to quantify the surface area of the P-g-C_3_N_4_/ZIF-67 composites (Figure 2b). The adsorption–desorption isotherms of P-g-C_3_N_4_/ZIF-67 exhibit a combination of type I and type IV profiles. The initial jump in adsorption capacity (P/P0 < 0.05) is caused by the filling of micropores in ZIF-67, followed by a gradual increase in uptake attributed to the presence of mesoporous space created by nanoparticle aggregates (Figure 2b). A hysteresis loop at 0.40 < P/P_0_ < 0.95 further confirms the existence of large porous structures. The calculated BET surface area of P-g-C_3_N_4_/ZIF-67 is 374.9 m^2^g^−1^, and the total pore volume of P-g-C_3_N_4_/ZIF-67 is 0.337 cm^3^g^−1^.

### 3.2. Electrochemical Behavior of Dopamine at the Surface of P-g-C_3_N_4_/ZIF-67/CPE

**Figure 3:**
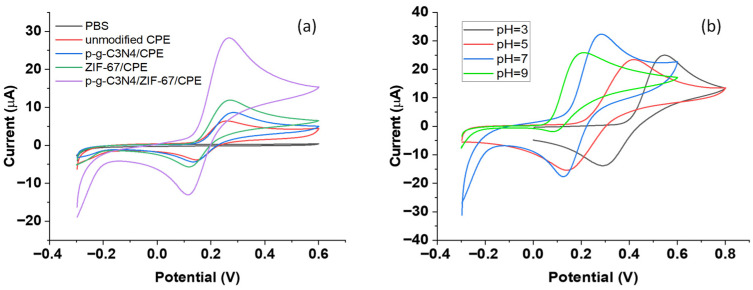
(**a**) The CVs of different electrodes in the presence of 500 μM dopamine in 0.1 M phosphate-buffered solution (pH = 7) with a scan rate of 100 mVs^−1^: (i) unmodified CPE, (ii) P-g-C_3_N_4_/CPE, (iii) ZIF-67/CPE, and (iv) P-g-C_3_N_4_/ZIF-67/CPE. The CV of the composite electrode in PBS alone is also provided for comparison. (**b**) Effect of pH on CVs of 500 μM dopamine at 100 mVs^−1^ in 0.1 M phosphate-buffered solution at a pH value of 3, 5, 7, and 9.

The cyclic voltammograms (CVs) of the P-g-C_3_N_4_/ZIF-67/CPE, P-g-C_3_N_4_/CPE, and unmodified CPE are shown in Figure 3a. At a scan rate of 100 mVs^−1^, the redox of dopamine in 0.1M phosphate-buffered solution (pH 7) produces an anodic peak on the forward scan (near ~0.26 V) and a cathodic peak (near ~0.12 V) on the reverse scan over all four carbon paste electrodes. For the electrode comprising P-g-C_3_N_4_/CPE or ZIF-67 alone, the oxidation peak current of dopamine is noticeably higher than on the unmodified electrode, which receives a significant boost upon the addition of both ZIF-67 and P-g-C_3_N_4_ to form the composite CPE (~5 times that of the unmodified electrode). In addition to the high surface area and rich active adsorption sites of ZIF-67, the Co centers within the ZIF-67 structure enable Co^2+^/Co^3+^ redox cycling, mediating rapid electron transfer from dopamine to the electrode surface for oxidation. The P-g-C_3_N_4_ further improves the overall conductivity of the composite electrode, thereby enhancing the electron-transfer rate at the P-g-C_3_N4/ZIF-67/CPE interface. The synergistic effect of P-g-C_3_N_4_ and ZIF-67 further leverages the electrocatalytic activity and sensing performance of the composite electrode.

### 3.3. Investigation of the pH Effect on the Electrochemical Behavior of Dopamine

To identify the best sensing performance, the electrochemical behavior of dopamine at the surface of C_3_N_4_/ZIF-67/CPE was examined in a pH range of 3.0 to 9.0 of the phosphate buffer solutions (0.1 M). As shown in Figure 3b, the CV curves of dopamine have a similar well-defined, quasi-reversible redox shape in acidic or neutral conditions (pH = 3–7). With increasing pH, the anodic peak current corresponding to dopamine oxidation rises until reaching its maximum at pH 7. The peak potential shifts to a less positive value, and the voltammogram becomes less reversible, which is clearly distorted at pH = 9. Such pH-dependent behaviors are caused by the involved multiple-step oxidation mechanism (Figure S1a): dopamine is first oxidized to dopamine-quinone reversibly by losing two electrons/photons, followed by the cyclization of the dopamine-quinone (Figure S1a). The cyclization process is irreversible, but could be suppressed in an acidic environment. When the electrochemical reaction occurs at pH 9, oxidation becomes favored (lower potential), followed by rapid cyclization, making the back-reduction less clean and the CV less reversible. Plotting the oxidation potential versus the solution pH displayed a linear relationship (Figure S1b). The slope of 58.3 mV pH^−1^ in the regression equation is very close to the theoretical Nernstian value of 59 mV pH^−1^, consistent with the 2H^+^/2e^−^ electrooxidation mechanism of dopamine. Nonetheless, pH 7 was selected in subsequent electrochemical tests, where the oxidation peak is strongest and proton-coupled electron transfer is efficient, while quasi-reversible redox is still maintained.

### 3.4. Investigation of the Effect of the Scan Rate on the Electrochemical Behavior of Dopamine

**Figure 4:**
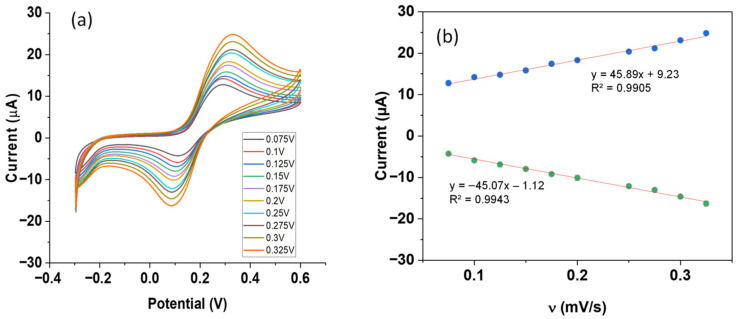
(**a**) CVs of 300 μM dopamine in 0.1 M phosphate-buffered solution (pH = 7) over the surface of P-g-C_3_N_4_/ZIF-67/CPE at a scan rate of 75–325 mVs^−1^. (**b**) Plots of the ***Ip*** with ***ʋ*** for the redox Peaks. Data points in red represent oxidation peak current, and those in green represent reduction peak current.

To further understand the electrooxidation process of dopamine at the surface of this new P-g-C_3_N_4_/ZIF-67/CPE, the scan rate effect of the cyclic voltammograms on the peak current of dopamine was investigated. The CVs of 300 μM dopamine in a phosphate-buffered solution (pH = 7) were recorded at a scan rate of 75–325 mV s^−1^. As shown in Figure 4a, the redox peak current rises with the increasing scan rate. The shape of the CV curves remains similar, while the oxidation peak potential shifts towards more positive values, and the reduction peak potential becomes more negative. Such rate-dependent peak separation confirms the quasi-reversible or irreversible behavior of the electrochemical reactions on this composite electrode. A well-fitted, linear relationship was observed between the peak current and the scan rate (***v***) in the entire scan range of 75–325 mV s^−1^, as shown in Figure 4b. In contrast, the plot of peak current versus the square root of the scan rate (***v***^1/2^) shows a similar trend only at slow scan rates, deviating at high scan rates (Figure S2). This observation indicates that the electrochemical reaction of dopamine at the P-g-C_3_N_4_/ZIF-67/CPE is adsorption-limited, a common feature of carbon- or MOF-based electrode modifiers.

### 3.5. Analytical Performance

**Figure 5:**
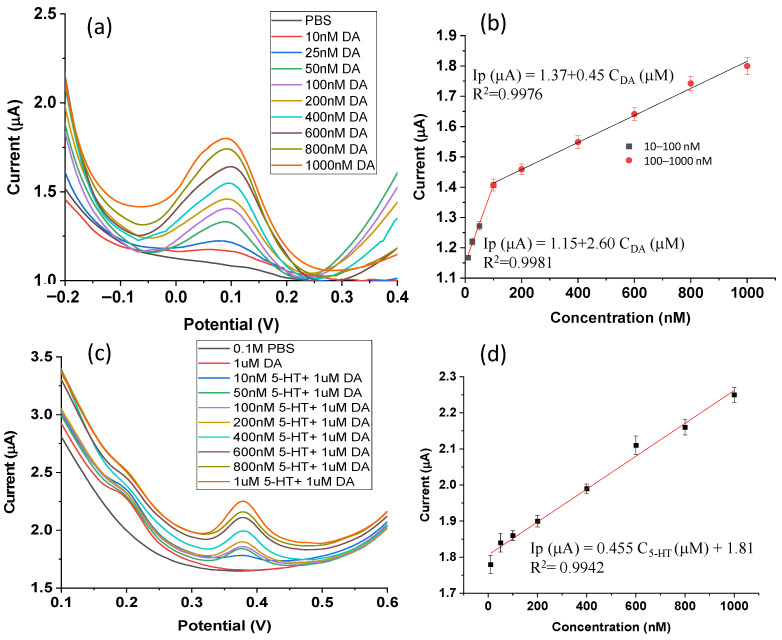
(**a**) DPVs of dopamine with different concentrations (0, 10, 25, 50, 100, 200, 400, 600, 800, and 1000 nM) in 0.1 M phosphate buffer (pH = 7); (**b**) the plot of peak current as a function of dopamine concentration over the range 10–1000 nM with two calibration curves generated: one for 10–100 nM and the other for 100–1000 nM, respectively; (**c**) DPV signals of a binary mixture with dopamine (1 μM) and serotonin at different concentrations (0, 10, 25, 50, 100, 200, 400, 600, 800, and 1000 nM) and (**d**) the calibration plot of peak current as a function of serotonin concentration and the calibration curve generated over the range 10–1000 nM.

As one of the most sensitive electrochemical methods, differential pulse voltammetry (DPV) was used to assess the sensitivity of the P-g-C_3_N_4_/ZIF-67/CPE sensor to dopamine. Under a pulse amplitude of 50 mV, a pulse width of 40 ms, and a scan rate of 60 mV s^−1^, the oxidation peak current of dopamine continuously rises in DPV scanning (from 1.17 µA to 1.80 µA) with the increase in dopamine concentration (10–1000 nM), as shown in Figure 5a. Two linear sensing concentration ranges were observed on the established calibration curve: 10–100 nM and 100–1000 nM (Figure 5b). At high concentrations of dopamine (100–1000 nM), the linear fit equation is ***Ip*** (μA) = 1.37 + 0.45 C_DA_ (μM) (R^2^ = 0.9976). At low concentrations (10–100 nM), the corresponding linear regression equation is ***Ip*** (μA) = 1.15 + 2.60 C_DA_ (μM) (R^2^ = 0.9981). This yields a sensitivity of 2.60 μA·μM^−1^ based on the slope method (Equation (1)). Further calculations from Equation (2) yield an ***LOD*** of 0.39 nM and a limit of quantity (***LOQ***) of 1.29 nM for this new P-g-C_3_N_4_/ZIF-67/CPE sensor for dopamine detection. Compared to some previously reported electrochemical sensors (Table 1), this work represents a significant advance.

### 3.6. Selectivity, Reproducibility, and Stability

To assess the selectivity of the composite electrochemical sensor, several major interfering compounds, including serotonin (5-HT), ascorbic acid (AA), and uric acid (UA), were added to the sample solutions. As shown in Figure 5c, the oxidation peak potentials of dopamine and serotonin are clearly different (DA: 0.17 V; 5-HT: 0.38 V). In a broad concentration range of serotonin (from 10 nM to 1000 nM), the interference of serotonin with the DPV signal of dopamine (1 μM) is negligible. In a series of binary mixtures (Figure 5c), the DPV peak current of serotonin rises systematically as its concentration increases from 10 nM to 1000 nM. Further analysis (Figure 5d) found a linear relationship, ***Ip***_5-HT_ = 0.455 C_5-HT_ (μM) + 1.81 (R^2^ = 0.9942) and an LOD of 167 nM. These results suggest that the P-g-C_3_N_4_/ZIF-67/CPE sensor can simultaneously detect both neurotransmitters (dopamine and serotonin) using DPV (Figure 5 & Supplementary Table S1).

**Figure 6:**
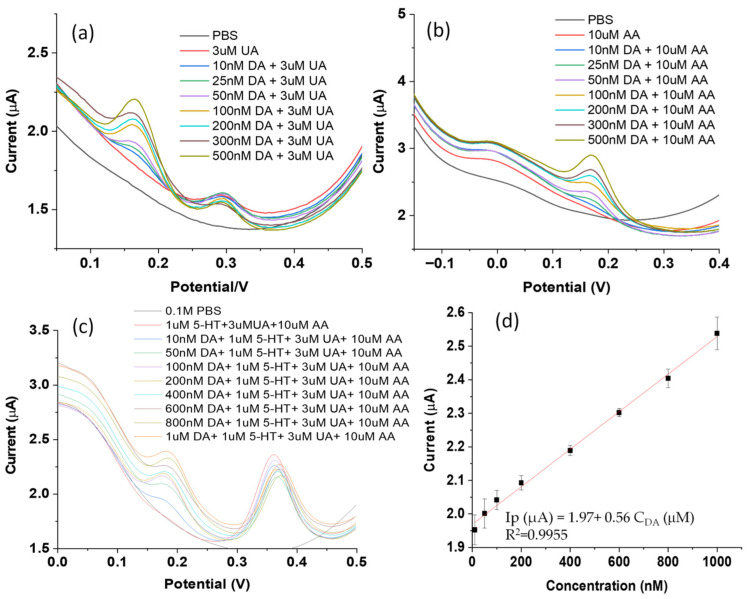
DPVs of dopamine with different concentrations (0, 10, 25, 50, 100, 200, 300, and 500 nM) in 0.1 M phosphate buffer (pH = 7) at the interference of uric acid of 3 μM (**a**) and ascorbic acid of 10 μM (**b**) in a binary mixture of all three (**c**), serotonin (1 μM), ascorbic acid (10 μM), and uric acid (3 μM), and the corresponding calibration curve (**d**) generated based on data in panel c.

As for other interference probes, such as uric acid (UA) and ascorbic acid (AA), their DPV peaks are also well separated from that of dopamine. In a binary system of DA + UA, DPV peaks of UA are located near 0.3 V, closer to the peak of serotonin (0.38 V) than dopamine (0.16 V), as shown in Figure 6a. The DPV peaks of ascorbic acid (AA) in the binary system (DA + AA) appear on the left side of the dopamine peaks, almost at 0 V, compared to 0.18 V for dopamine (Figure 6b). When all three interference molecules are presented simultaneously, the oxidation peak of dopamine stays at 0.18 V in this ternary system, well separated from that of interference compounds (uric acid of 3 μM, ascorbic acid of 10 μM, and serotonin of 1 μM), as shown in Figure 6c. The DPV peak of UA largely overlaps with the serotonin peak near 0.38 V, while that of AA slightly shifts to ~0.05 V. In other words, the presence of these interference compounds barely affects the DPV peak position of dopamine. These results verify that the oxidation of dopamine at the P-g-C_3_N_4_/ZIF-67 composite electrode is independent of many important interfering biomolecules. A reasonably linear relationship between the DPV peaks and a broad concentration range of dopamine (0–1000 nM) was observed, as shown in Figure 6d. The linear fit equation is ***Ip*** (μA) = 1.97 + 0.56 C_DA_ (μM) (R^2^ = 0.9955). These results demonstrate the selective detection of dopamine with this P-g-C_3_N_4_/ZIF-67/CPE electrochemical sensor, suggesting its potential for real-sample sensing.

To verify the reproducibility of this composite sensor, DPV tests of a solution containing 200 nM dopamine were performed on three independently prepared P-g-C_3_N_4_/ZIF-67/CPEs. Their similar peak current readings (with a standard deviation of less than ±0.02) demonstrate the acceptable reproducibility of this sensor (Figure S4). As for anti-fouling tests, a 1.39% drop in peak current was observed after three hours (Figure S5 & Supplementary Table S2). Together with data from multiple scan cycles (Figure S3), the P-g-C_3_N_4_/ZIF-67/CPE exhibits excellent operational stability. The long-term storage tests were conducted by measuring dopamine daily using DPV for 7 days, with the composite electrodes kept at 4 °C when not in use. The results show that the prepared P-g-C_3_N_4_/ZIF-67/CPE sensor retains 95% of its initial signal after a week-long test, demonstrating its good long-term storage stability (Figure S6 & Supplementary Table S3).

### 3.7. Analytical Application

To evaluate the sensing feasibility of this P-g-C_3_N_4_/ZIF-67/CPE in real applications, dopamine was spiked in urine samples. After a 100-fold dilution of collected human urine in PBS (pH = 7), dopamine was added to prepare testing solutions at final concentrations of 10–100 nM. The dopamine/urine solutions were then measured using the P-g-C_3_N_4_/ZIF-67/CPE electrochemical sensor, and the dopamine concentration was determined from the pre-established calibration curve (Figure 6d). The corresponding recovery rate was then calculated from these results. As shown in Table 2, for urine samples spiked with dopamine at concentrations of 10–100 nM, the recovery rate varies from 99.2% to 104.8%, with relative standard deviations (RSDs) of 0.7% to 2.1%. These data confirm the excellent performance of this P-g-C_3_N_4_/ZIF-67 sensor in detecting dopamine in real samples, supporting its use in future practice.

### 3.8. Discussions

**Figure 7:**
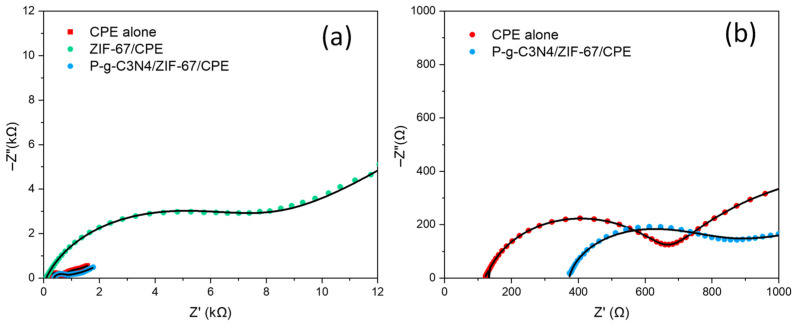
EIS for (**a**) all three electrodes (CPE, ZIF-67/CPE, and P-g-C_3_N_4_/ZIF-67/CPE) in 5 mM [Fe(CN)_6_]^3−/4−^, and (**b**) enlarged plots of only CPE and P-g-C_3_N_4_/ZIF-67/CPE from panel a.

In this study, a new P-g-C_3_N_4_/ZIF-67 composite was used to modify carbon paste electrodes. Inside the electrode modifier, ZIF-67 is expected to provide numerous active sites needed for electrochemical reactions due to the microporous structure within its crystal, while P-g-C_3_N_4_ enhances overall electrical conductivity. The P-g-C_3_N_4_/ZIF-67 composites also carry additional mesoporous pathways to further improve dopamine diffusion to enrich its presence near the redox reaction sites. The reactions between Co^2+^ and Co^3+^ within ZIF-67 further pair with the oxidation/reduction processes of dopamine molecules to accelerate overall electron transfer rates. Their syngenetic effect boosts the electrochemical reaction efficiency over the P-g-C_3_N_4_/ZIF-67 electrode and its sensitivity to dopamine. As shown in Figure 3a, the redox peak (as well as the CV area) of P-g-C_3_N_4_/ZIF-67, under the same scan rate, was found to be the largest among those of all four CPEs. This confirms that the highest concentration of dopamine molecules is present near the P-g-C_3_N_4_/ZIF-67 electrode. Although it is attributed to both ZIF-67 and P-g-C_3_N_4_ in the composite electrodes, ZIF-67 likely contributes more than P-g-C_3_N_4_ to the high redox peak current (and sensitivity), given the fact that the redox peaks (and the CV area) of ZIF-67/CPE are also higher than those of P-g-C_3_N_4_/CPE. The roles of P-g-C_3_N_4_ are believed to be largely tied to the reduction in internal resistance to electron and/or proton transport. To verify this hypothesis, EIS tests were done on CPE alone, CPE with ZIF-67, and CPE with P-g-C_3_N_4_/ZIF-67. As shown in Figure 7, the Nyquist plot of the CPE with P-g-C_3_N_4_/ZIF-67 (blue dots) exhibits a charge-transfer resistance of 687 Ω, comparable to that of the CPE alone (690 Ω). In contrast, the charge-transfer resistance of the CPE with only ZIF-67 (green dots) is more than 10 times larger (7360 Ω). This suggests that the addition of P-g-C_3_N_4_ significantly offsets the nonconductive drawback of ZIF-67 in the composite electrode, reduces its charge-transfer resistance, and thereby enhances the sensing signal of the fabricated P-g-C_3_N_4_/ZIF-67 sensor.

## 4. Conclusions

In summary, a new electrochemical sensor was prepared by combining phosphorus-doped graphitic carbon nitride with a cobalt-based metal–organic framework (P-g-C_3_N_4_/ZIF-67). The successful synthesis of the composite electrode material was first confirmed with SEM, XRD, and EDS images or spectra. The morphology and surface properties of the received P-g-C_3_N_4_/ZIF-67 were further revealed by SEM, BET, and FTIR measurements. Benefiting from the advantages of this new electrode modifier, namely large surface area, porous structure, and high electrical conductivity, a limit of detection of 0.39 nM was found when measuring dopamine within a linear sensing range of 10–100 nM. The new dopamine sensor is selective, reproducible, and stable in the presence of other neurotransmitters or interfering molecules. Dopamine (10–100 nM) spiked in urine samples was successfully quantified with a recovery rate of 99.2–104.8% and RSDs of 0.7% to 2.1%. These promising results suggest potential practical use of this new P-g-C_3_N_4_/ZIF-67 electrochemical sensor for future neutral-sensing applications.

## Figures and Tables

**Table 1:** Comparison of the performance of various electrodes for dopamine detection.

Electrode Materials	Linearity	*LOD* (Dopamine)	Ref
NanoSnO_2_/MWCNTs/CPE	0.3–50 μM	30 nM	[40]
Nafion/AuNPs/AzA/MWCNTs	0.5–10 μM	14 nM	[41]
Fe_3_O_4_/polyprrole/reduced graphene oxide	0 to 100 μM	63 nM	[42]
Nafion-BDUNCD-MWCNT (with Nitric Acid Treatment)	1 to 50 μM	1.78 nM	[43]
Au/CNTPCA/GCE	100 to 12 μM	1 nM	[44]
MWCNT-DHP film-coated GCE	0.05–5 μM	11 nM	[45]
SWCNT/carbon-fiber electrodes	5.0–120.6 μM	30 nM	[46]
MWCNT/PSVM/Au/GCE	0.1 to 200 μM	56 nM	[47]
MWCNT/graphene oxide (GO) GCE	0.2 to 400 μM	22 nM	[48]
CNT/MWCNT Polymer Micropillar Array	1 to 50 nM	0.77 nM	[49]
ZIF-8	0.05–0.5 μM	195 nM	[50]
Ag-ZIF-67/GCE	0.1–100 μM	50 nM	[51]
g-C_3_N_4_/MWCNTs/GO	4–200 μM	220 nM	[52]
Fe-porous carbon nanosheets from Fe-MOF	0.5–100 μM	15.54 nM	[53]
Au@Cu-metal–organic framework	10–1000 μM	3.40 μM	[54]
P-g-C_3_N_4_/ZIF 67/CPE	10 to 100 nM	0.39 nM	This work

Abbreviations: GCE: glass carbon electrode; AuNPs: gold nanoparticles; AzA: Aza-Polycyclic Aromatic Hydrocarbons; MWCNTs: multi-wall carbon nanotubes; BDUNCD: boron-doped ultra-nanocrystalline diamond; PCA:phenazine-1-carboxylic Acid; DHP: di-hexadecyl hydrogen phosphate; PSVM: poly(vinyl benzyl thymine-co-styrene-co-maleic anhydride); Au: gold; CNT: single-wall carbon nanotubes; Ag: silver; GO: graphene oxide.

**Table 2:** Determination of dopamine in urine samples (***n*** = 5) using P-g-C_3_N_4_/ZIF-67/CPE.

Sample	Added (nM)	Found (nM)	Recovery (%)	RSD (%)
Dopamine	10	9.9 ± 4.0	99.2 ± 0.4	2.1
	25	25.6 ± 9.4	102.3 ± 0.4	1.8
	50	49.8 ± 4.2	99.7 ± 0.1	0.7
	75	74.2 ± 9.6	98.9 ± 0.1	1.6
	100	104.8 ± 10.6	104.8 ± 0.1	1.7

## Data Availability

The data presented in this study is available on request from the corresponding author.

## Supplementary Materials

The following supporting information can be downloaded at: https://www.mdpi.com/article/10.3390/bios16040224/s1. Table S1: Sensitivity parameters of the P-g-C_3_N_4_/ZIF-67/CPE from the DPV tests on dopamine and serotonin. Table S2: Fouling of the P-g-C_3_N_4_/ZIF-67/CPE during dopamine tests. Table S3: Long-term stability test of the P-g-C_3_N_4_/ZIF-67/CPE from DPV dopamine sensing. Figure S1. (a) The electrochemical oxidation mechanism for dopamine; (b) the relationship between the oxidation peak potential (Epa) and pH. Figure S2. Plots of the redox peak current (Ip) with ʋ^1/2^ for CV scanning of 100 µM dopamine in 0.1 M phosphate buffer solution (pH = 7) over the surface of P-g-C_3_N_4_/ZIF-67/CPE at a scan rate of 75–325 mVs^−1^. Figure S3. CVs of 10 µM dopamine in 0.1 M phosphate buffer solution (pH = 7) over the surface of the P-g-C_3_N_4_/ZIF-67/CPE electrode over cycles 1, 2, and 10. Figure S4. The DPVs of three different P-g-C_3_N_4_/ZIF-67/CPE sensors on 200 nM dopamine in 0.1 M phosphate buffer solution (pH = 7). Figure S5. The DPVs of the P-g-C_3_N_4_/ZIF-67/CPE sensor on 500 nM dopamine in 0.1 M phosphate buffer solution (pH = 7), every 20 min for 200 min. Figure S6. The long-term stability tests of the P-g-C_3_N_4_/ZIF-67/CPE sensors with a week-long DPV scan of 100 nM dopamine in 0.1 M phosphate buffer solution (pH = 7).
