# Supplementary material for: Electrochemical Sensing of Dopamine with P-g-C3N4/ZIF-67/CPE Composite Electrodes
Source: Biosensors (Basel). 2026 Apr 18;16(4):224. doi: 10.3390/bios16040224 (PMC13115184; doi:10.3390/bios16040224)
Supplement: Supplementary file 1 [file biosensors-16-00224-s001.zip › biosensors-4211531-supplementary.pdf]

Electrochemical Sensing of Dopamine with P-g-C_3N_4/ZIF-67/CPE Composite Electrodes

Yan Deng [1,†], Yixin Liao [1,†], Teresa Murray [2] and Shengnian Wang [1,2,*]

[1] Institute for Micromanufacturing, Louisiana Tech University, Ruston, LA 71272, USA; dya006@latech.edu (Y.D.); yliao@latech.edu (Y.L.)

[2] Center for Biomedical Engineering and Rehabilitation Sciences, Louisiana Tech University, Ruston, LA 71272, USA; tmurray@latech.edu

* Correspondence: swang@latech.edu; Tel.: +1-318-257-5125
† These authors contributed equally to this work.

Keywords: metal–organic framework (MOF); ZIF-67; carbon nitride; electrochemical sensor; dopamine

Supplementary Table S1. Sensitivity parameters of the P-g-C_3N_4/ZIF-67/CPE from the DPV tests on dopamine and serotonin

	RSD (%)								
	10 nM	25 nM	50 nM	100 nM	200 nM	400 nM	600 nM	800 nM	1 µM
dopamine	0.29	0.91	1.05	1.39	1.22	1.38	1.30	1.37	1.55
serotonin	1.42	-	1.40	0.76	0.87	0.64	1.21	0.98	0.92

Supplementary Table S2. Fouling of the P-g-C_3N_4/ZIF-67/CPE during dopamine tests

	2/3 hr	1 hr	4/3 hr	5/3 hr	2 hr	7/3 hr	8/3 hr	3 hr
Changes (%)	0.38	0.51	0.71	0.88	1.05	1.16	1.30	1.39

Supplementary Table S3. Long-term stability test of the P-g-C_3N_4/ZIF-67/CPE from DPV dopamine sensing

Time	Fresh	1-day	2-day	3-day	4-day	5-day	6-day	7day
Changes (%)	-	0.11	0.20	0.25	0.42	0.39	0.41	0.24

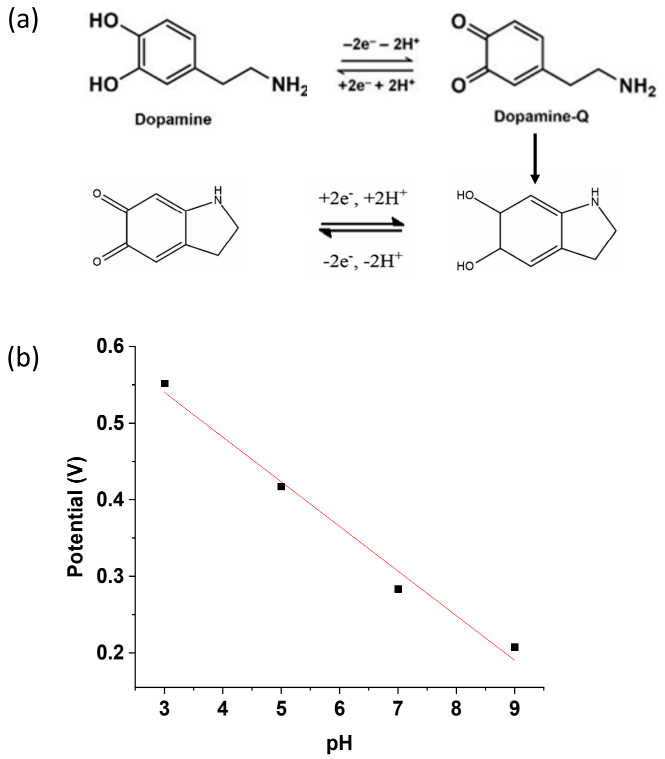

Figure S1. (a) The electrochemical oxidation mechanism for dopamine; (b) the relationship between the oxidation peak potential (Epa) and pH.

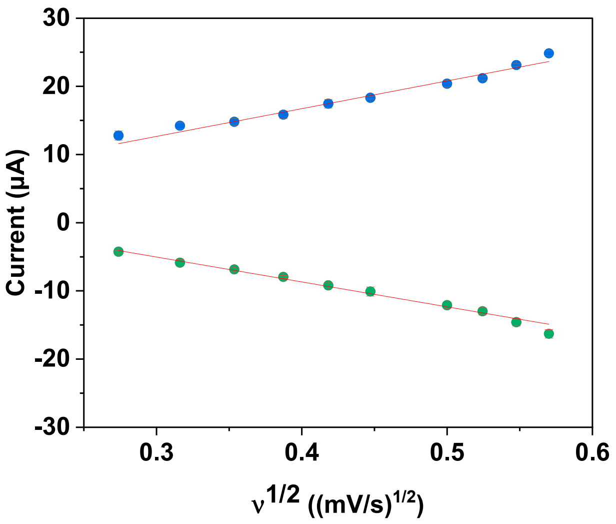

Figure S2. Plots of the redox peak current (Ip) with $\upsilon^{1/2}$ for CV scanning of 100 μM dopamine in 0.1 M phosphate buffer solution (pH=7) over the surface of P-g-C_3N_4/ZIF-67/CPE at a scan rate of 75-325 mVs^{-1}.

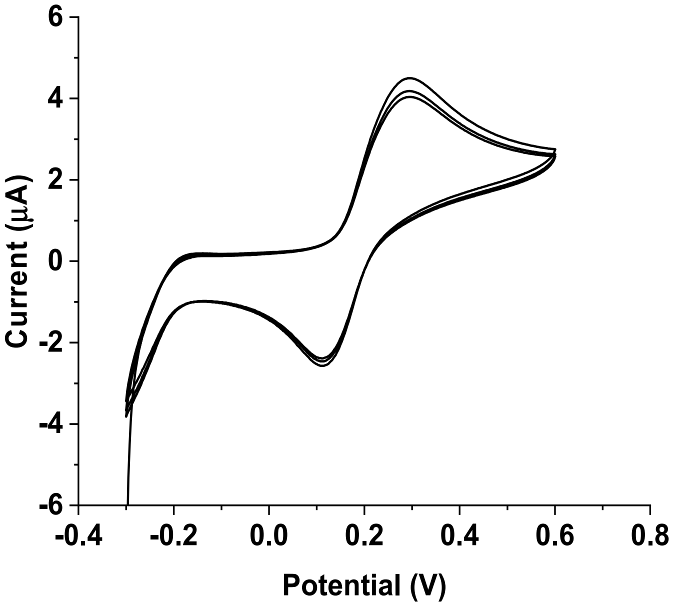

Figure S3. CVs of 10 µM dopamine in 0.1 M phosphate buffer solution (pH=7) over the surface of the P-g-C_3N_4/ZIF-67/CPE electrode over cycles 1, 2, and 10.

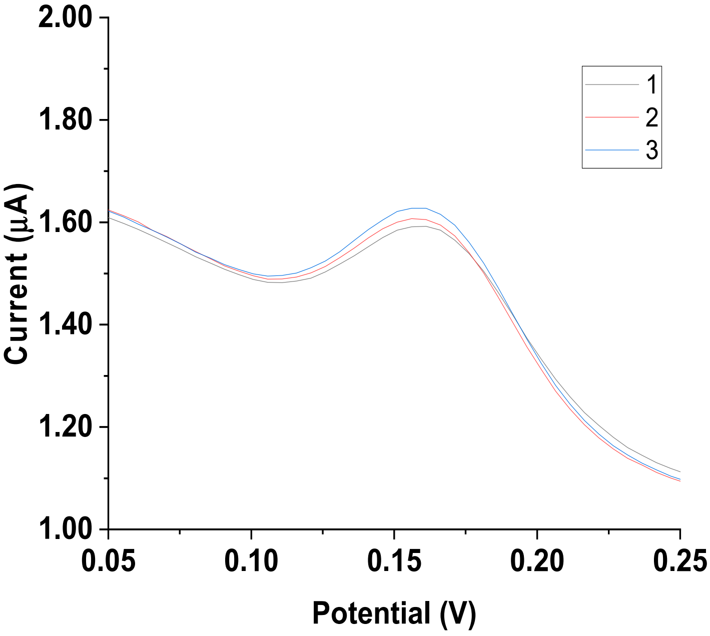

Figure S4. The DPVs of three different P-g-C_3N_4/ZIF-67/CPE sensors on 200 nM dopamine in 0.1 M phosphate buffer solution (pH=7).

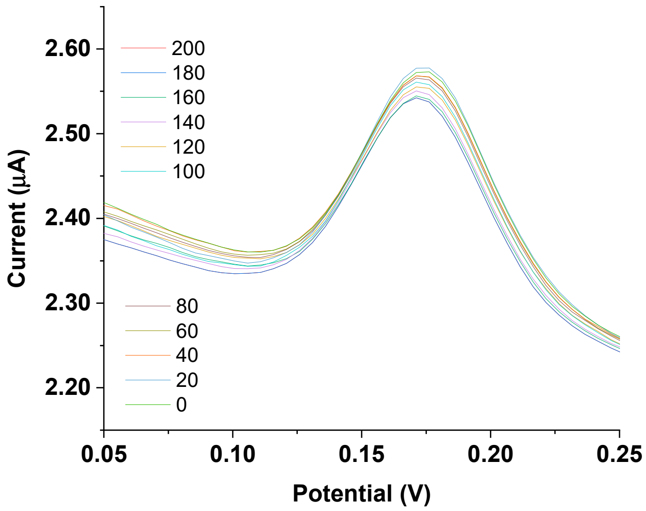

Figure S5. The DPVs of the P-g-C_3N_4/ZIF-67/CPE sensor on 500 nM dopamine in 0.1 M phosphate buffer solution (pH=7), every 20 minutes for 200 minutes.

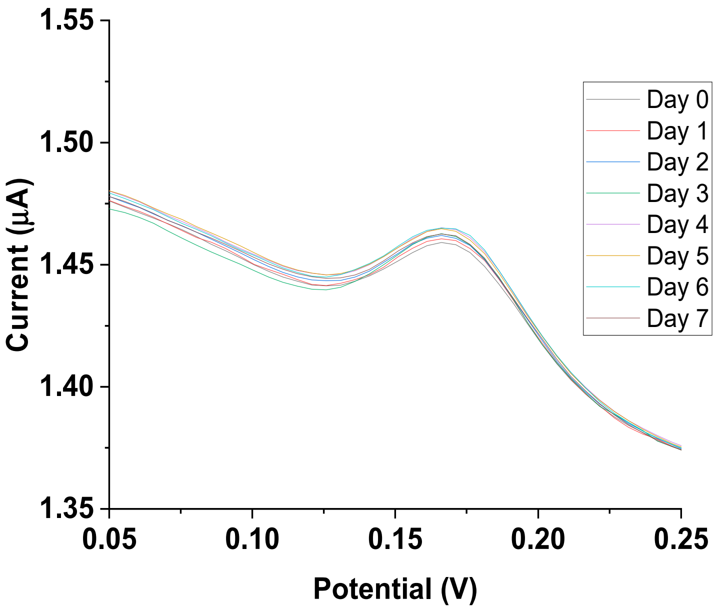

Figure S6. The long-term stability tests of the P-g-C_3N_4/ZIF-67/CPE sensors with a week-long DPV scan of 100 nM dopamine in 0.1 M phosphate buffer solution (pH=7).
